# Stress and Depression in Relation to Functional Health Behaviors in African American Patients with Systemic Lupus Erythematosus

**DOI:** 10.4172/2161-1149.S4-005

**Published:** 2014-11-07

**Authors:** Edith M. Williams, Larisa Bruner, Megan Penfield, Diane Kamen, James C. Oates

**Affiliations:** 1 Institute for Partnerships to Eliminate Health Disparities, University of South Carolina; Columbia, SC, USA; 2 Office of Public Health Practice, University of South Carolina; Columbia, SC, USA; 3 Institutional Assessment and Compliance, University of South Carolina; Columbia, SC, USA; 4 Division of Rheumatology and Immunology, Department of Medicine, Medical University of South Carolina; Charleston, SC, USA

**Keywords:** Systemic lupus erythematosus, Stress, Psychological distress, Coping, Depression, Health behaviors

## Abstract

**Objective:**

While increased psychological distress in SLE has been clinically and empirically reported, the relationship between emotional distress, treatment adherence, and disease activity are complex and even more unclear in African American lupus patients. In an effort to elucidate this phenomenon in these patients, this exploratory study aimed to investigate relationships between stress, depression, and various health behaviors in this group.

**Methods:**

Thirty patients invited to participate in this study were African American systemic lupus erythematosus (SLE) patients attending rheumatology clinics at the Medical University of South Carolina (MUSC). This study was part of a larger interventional pilot study, the Balancing Lupus Experiences with Stress Strategies (BLESS) study, that included a comprehensive battery of psychosocial, quality of life, and behavior change measures.

**Results:**

When looking at the association between anxiety/stress and functionality, levels of reported stress had strong effects upon functionality, especially between health distress and functionality. When looking at the association between depressive symptoms and functionality, depressive symptoms had moderate effects upon social/role limitations and nights spent in the hospital.

**Conclusion:**

Not only did the larger pilot project demonstrate significant reductions in stress and depression as a result of workshop participation; this nested study also showed that those improvements were positively associated with improved health behaviors. These results could have implications for developing interventions to improve disease experience and quality of life in SLE patients with stress and depression.

## Introduction

Psychological stress has been implicated in the development and severity of autoimmune diseases like SLE [1]. Studies reveal that lupus patients have a 50% chance of developing some form of psychological distress either because of direct central nervous system involvement, compounding systemic complications, effects of treatment, or adjustment related to chronic illness [2]. Research conducted using the Composite International Diagnostic Interview and the Systemic Lupus Activity Questionnaire analyzed three hundred twenty-six women with lupus. The study found that 65% of these patients received a lifetime mood or anxiety diagnosis. Other disorders that were prevalent in these women were major depressive disorder (MDD) found in 47%, specific phobias found in 24%, panic disorder in 16%, obsessive-compulsive disorder in 9%, and bipolar I disorder in 6% [3].

The relationship between psychological problems, such as depression, and maladaptive health behaviors, such as poor adherence to therapeutic regimens and failure of patients to keep scheduled appointments, in various medical populations has been well documented [4–9]. Further, non-adherence with treatment recommendations and poor appointment-keeping behaviors have been associated with worse outcome in numerous clinical disorders [10–12]. A handful of studies and some clinical observations have investigated adherence, including appointment-keeping behavior, and its effects on disease outcome in patients with SLE. Karlson et al. for example, studied the factors associated with disease damage and disease activity in a cross-sectional study of 200 SLE patients; adherence failed to account for either of these outcomes [13]. Others have found higher rates of emergency consultation, hospitalization, and renal damage in noncompliant patients [14–16].

This phenomenon appears to be even more pronounced in African Americans [17–20]. For example, Petri et al. found that physicians rated African-Americans as less globally adherent than whites (43.5% versus 66.3% adherent, respectively). They observed that African American patients with SLE had poorer renal outcomes than white patients, and this difference was related to increased hypertension and poorer treatment adherence among the African American patients [14]. Another study conducted by Uribe and colleagues (2004) to determine the baseline factors predictive of poor adherence with follow-up study visits in a longitudinal multiethnic lupus cohort study found that non-compliant patients were more likely to be of African American ethnicity and have longer disease duration and greater disease activity as assessed by the physician than the compliant patients [17].

To address the reasons for medical non-adherence among African American patients, Mosley-Williams and colleagues examined whether African-American and white women with SLE differed in potential barriers to adherence, rates of adherence to taking medication and attending clinic visits, and how identified barriers related to actual adherence behaviors for each ethnic group separately [18]. Researchers found that barriers related to negative effect, including depression, medication concerns, and physical symptoms, were associated with non-adherence among African-Americans [18].

There are multiple potential mechanisms by which every day and lifetime stress may adversely affect disease pathology in African- American lupus patients. Previous studies have observed that improvements in biological markers of stress, psychological function, and physical function for medical patients can reduce costs for medical care [21] and result in fewer emergency room visits, hospitalizations and nights in the hospital [22,23]. Given such findings, the aims of this study were to investigate relationships between stress, depression, and various health behaviors in African Americans with SLE, who are at highest risk for the disease, in an effort to understand the role of psychological well-being on health behaviors that directly impact disease severity in the most affected population.

For the purposes of this study, “stress” was examined according to self-reported “psychological distress” and other quality of life indicators. “Functional health behaviors” were investigated as a mix of health care utilization, perceptions of illness impact, and coping behaviors.

## Patients and Methods

### Subjects

Patients invited to participate in this study were African American systemic lupus erythematosus (SLE) patients attending rheumatology clinics at the Medical University of South Carolina (MUSC). All SLE patients met at least four components of the 1997 ACR revised criteria for SLE [24], were 18 years of age or older, and had not previously participated in a self management program. The total number of individual patients with SLE followed by clinicians at MUSC was 1,121 between 2009 and 2012. The total number of new patients with SLE seen by clinicians at MUSC between 2011 and 2012 was 176, of which 61% were African-American and 88% were female. Patients invited to participate in this study were lupus patients participating in a longitudinal observational web-based SLE Database at MUSC. There were 402 patients with lupus enrolled in the Database during enrollment in this study. Patients in the Database were characterized longitudinally for disease activity and quality of life. The vast majority of subjects have had serum/urine/DNA/RNA specimens collected and stored at −80°C. As part of the informed consent process, participants agreed to future re-contact regarding other research studies. MUSC’s SLE cohort is geographically diverse, representing more than 60 South Carolina and North Carolina counties. Of the 402 patients with lupus, 336 were African-American, and 218 of whom were Gullah African-American from the Sea Islands of South Carolina and Georgia. Recruitment efforts were attempted among all 336 African-American database participants, and the first thirty patients to respond and meet eligibility criteria were selected for participation in the current pilot study. This sub-set of database participants was comparable to the larger cohort of African-Americans with lupus at MUSC, with regard to female: male ratio, age, and other sociodemographic characteristics.

### Measures

This study was part of a larger interventional pilot study, the Balancing Lupus Experiences with Stress Strategies (BLESS) study [25,26], that included a comprehensive battery of psychosocial, quality of life, and behavior change measures [21–23,27–35]. To investigate relationships between stress, depression, and various health behaviors, the analyses for this study utilized the following measures:

Psychosocial stress was assessed by five validated measures. The State-Trait Anxiety Inventory (STAI) was initially conceptualized as a research instrument for the study of anxiety in adults [27]. It is a self-report assessment device which includes separate measures of state and trait anxiety. STAI is a 20 item 4 point likert scale, where responses range from “not at all” to “very much so” (where “0” represents no likelihood of experiencing a form of anxiety and “4” represents high likelihood of experiencing anxiety). The Arthritis Self-Efficacy Scale pain and other symptoms sub-scale [28] consists of 11 items designed to measure confidence in one’s ability to manage the pain, fatigue, frustration, and other aspects of disease; it was recorded in previous investigations to reflect lupus rather than arthritis [36]. The scale consists of ranges from 0–100, with three break points of “very uncertain” (0), “moderately uncertain” (50), and “very certain” (100). The Perceptions of Racism Scale is a 20-item self-report inventory concerning medical and lifetime experiences of discrimination [29,30]. The Perceptions of Racism Scale is on a 7 point likert scale whereas “several times a day” (0) represents the likelihood that an individual experiences racism more frequently and “never” (0) the opposite measurement effect. Health distress was assessed using a modified version of the Medical Outcomes Study (MOS) health distress scale, adapted by the Stanford Patient Education Research Center [31]. This scale consists of 3 questions in a likert format, whereas “none of the time” (0) indicates a patient does not experience health distress and “all of the time” (5) representing the opposite. The Beck Depression Inventory (BDI) is a 21-question multiple-choice self-report inventory for measuring the severity of depression [32], in a 4 point likert scale format (lower end of the scale representing normality in emotional coping and the upper end representing extreme depression). It is composed of items relating to symptoms of depression such as hopelessness and irritability, cognitions such as guilt or feelings of being punished, as well as physical symptoms such as fatigue, weight loss, and lack of interest in sex.

Quality of life was assessed using two instruments that describe a spectrum of quality of life outcomes. The LUP-QOL incorporates the Medical Outcomes Study (MOS) Short Form 36 Health Survey (SF-36) and the Functional Assessment of Chronic Illness Therapy-Fatigue (FACIT-F), which are reliable and valid instruments that are frequently used in quality of life studies of persons with lupus [31,33,34]. The questionnaire includes questions pertaining to physical function, role function, social function, mental health, health perception and pain.

Behavior Change was assessed using Stanford Patient Education Research Center Questionnaires assessing medical outcomes such as hospital visits, illness intrusiveness, and use of stress management techniques [21–23,35]. These are behavior change scales, modified from the Medical Outcomes Study, to determine if participants are practicing cognitive stress reduction (pain reduction) and non-cognitive (mental stress management/relaxation) techniques. These scales also assess whether key behaviors concerning communicating with health care providers and health care utilization have changed.

### Statistical analyses

Thirty participants were randomly assigned to intervention and control groups. Table 1 shows that all participants were African American, 28 were female, and more than half were either attended trade school or college. Two of the participants assigned to the intervention group did not attend any intervention sessions and were eliminated from all post-intervention analyses. In addition, several participants did not complete post-intervention questionnaires and were also excluded from analyses. Therefore, data were analyzed on 30 participants at baseline, 25 (N=13 for control group and N=12 for intervention group) at post-intervention, and 22 (N=12 for control group and N=10 for intervention group) at four months postintervention. "Per-protocol" (or the elimination of any participants that did not complete treatment) rather than "intent-to-treat" (inclusion of all participants regardless of whether they completed treatment) analyses were undertaken due to missing survey data at specified data collection points from most of the excluded participants. Intent-to-treat analyses would have been suitable if excluded participants had completed the study (i.e., provided responses at specified data collection points); even if they did not receive treatments they should have (i.e., completed intervention sessions).

Given participant dropout and the exploratory nature of this study, statistical tests were uncertain due to violation of assumptions and low power. Descriptive statistics related to stress, depression, and various health behaviors were summarized using SAS (Cary NC) statistical software and reported along with measures of power and correlation [37]. Cohen’s effect sizes (d) were computed as a measure of power using the software program g-power. Typically, Cohen’s d is only reported as a unidirectional statistic ranging from 0 to infinity (typically not above 2 or 3). The magnitude of Cohen’s d is similar to that of Pearson’s r, wherein a value of .2 indicates small effect, .5 indicates medium effect, and .8 indicates large effect. Reporting Cohen’s d was chosen for this study because it is independent of sample size, unlike statistical methods such as t-tests. In this case, effect sizes show the actual magnitude of the difference-not just how likely the results are to have occurred by chance.

## Results

### Coping

**Figure 1:**
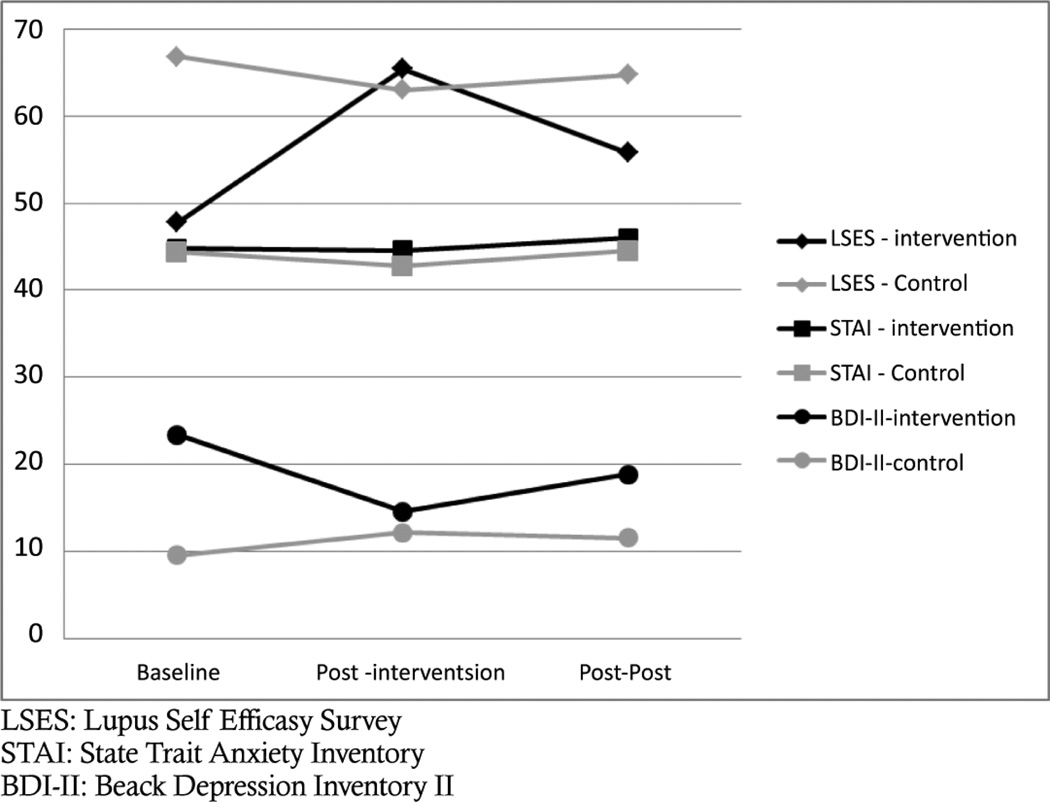
Changes in Coping among Intervention and Control Participants (N=30).

**Figure 2:**
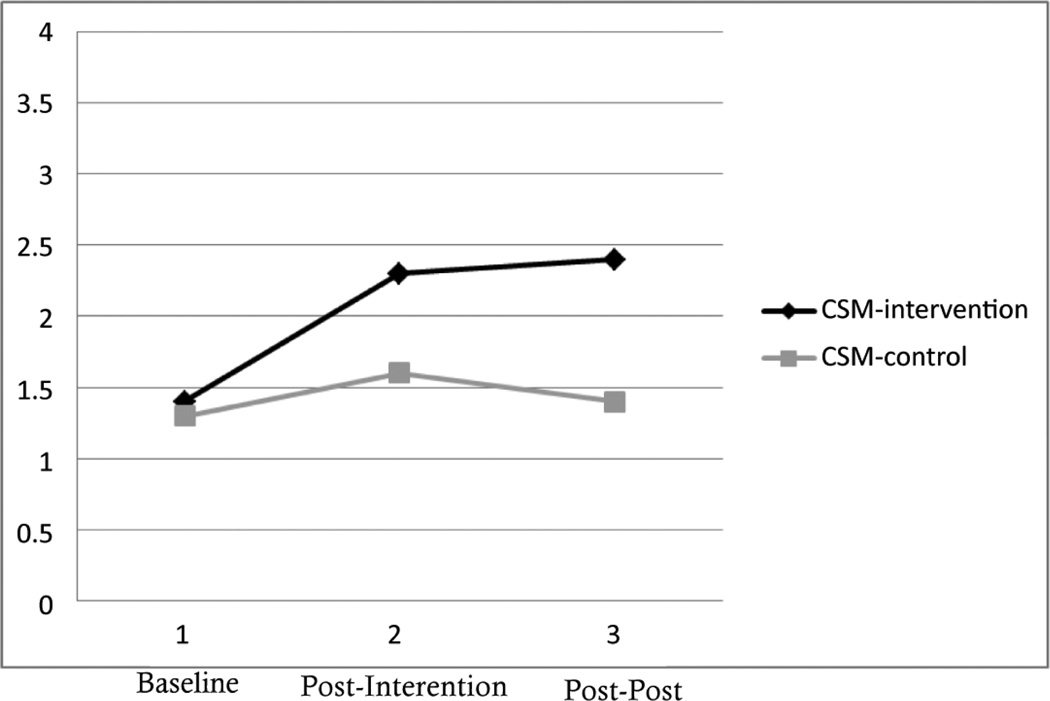
Changes in Cognitive Symptom Management (CSM) among Intervention and Control Participants (N=30).

Compared to baseline measurements of coping, participants in the intervention group displayed improvement in self-reported coping compared to controls over time, especially between baseline and post-intervention. As can be seen in Figure 1, individuals in the control group held consistent scores on the Lupus Self-Efficacy survey (LSES), while participants in the intervention group reported more self-efficacy at post-intervention and post-post intervention when compared to baseline. Similarly, participants in the control group held constant scores on the Cognitive Symptom Management (CSM) survey, while scores for participants in the intervention group increased first at post-intervention and then again slightly at post-post intervention (Figure 2).

When looking at measurements of power, participation in the workshops had a large effect upon coping as measured by the LSES (d=0.85) at post-intervention. Moderate effects were witnessed between workshop participation and the CSM and workshop participation and the LSES at post-post intervention (Table 2).

In terms of correlation, as can be seen in Table 3, coping had a moderate effect upon functionality as measured by reported pain, visits to the hospital, reported social/role limitations and exercise behaviors. Specifically, when looking at scores on the LSES, improved self-efficacy was moderately associated with less social/role limitations (*r* =−0.36), visits to the hospital (*r* =−0.33) and pain (*r* =−0.40*). There was also a negative moderate relation between scores on the CSM and social/role limitations (*r* =−0.38) as well as a positive association between CSM scores and reported exercise (*r* =0.46*).

### Anxiety and stress

**Figure 3:**
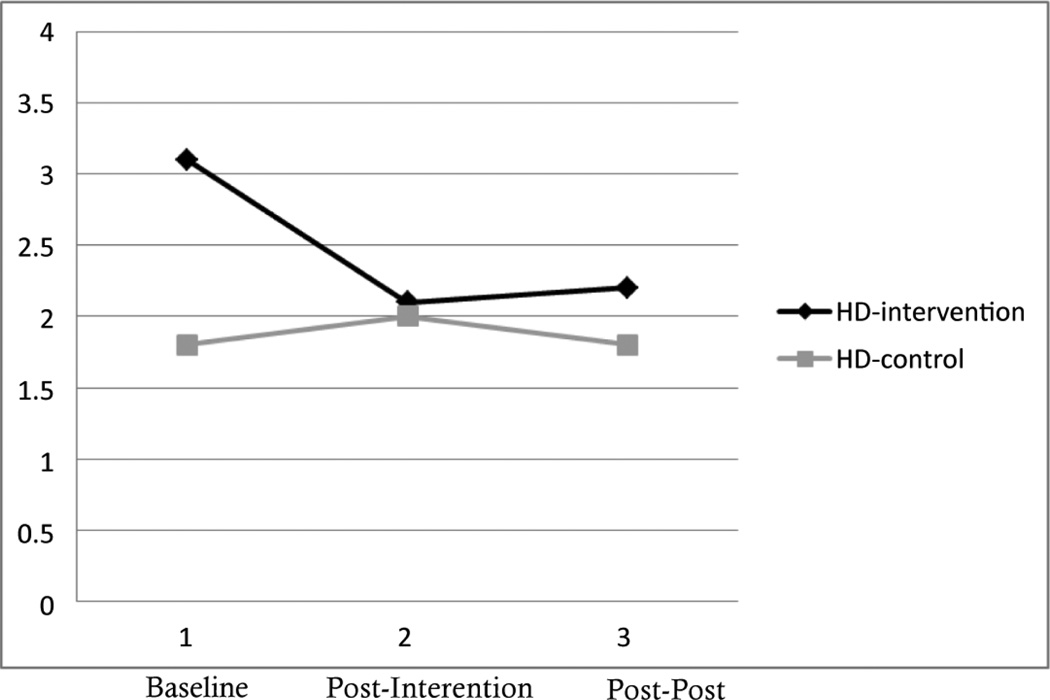
Changes in Health Distress (HD) among Intervention and Control Participants (N=30).

The effects of the intervention on anxiety and stress were mixed. Although participants in the intervention group reported similar levels of stress on the State-Trait Anxiety Inventory (STAI) between baseline and post-post intervention (Figure 1), they reported decreases in health distress (Figure 3). As with measures of coping, individuals in the control group held constant scores on measures of anxiety and health distress.

As can be seen in Table 2, effect sizes for workshop participation and health distress were large at both post-intervention (*d* =0.94) and post-post intervention (*d* =0.73). However, there was only a small effect between workshop participation and scores on the STAI at either time point.

When looking at the association between anxiety/stress and functionality, levels of reported stress had strong effects upon functionality, especially between health distress and functionality. More health distress was strongly associated with more social/role limitations (*r* =0.50*) and more pain (*r* =0.66**) (Table 3). Also, as levels of reported anxiety increased (as measured by the STAI), reported levels of illness intrusiveness also improved (*r* =0.048*). In addition, improved scores on the STAI were associated with less visits to the physician (*r* =−0.45*) and more reported exercise (*r* =0.43*).

### Depression

Consistent with all other findings, participants in the control group exhibited minimal differences in scores on the Beck Depression Inventory II (BDI-II) between baseline and post-post intervention (Figure 1). However, participants in the intervention group displayed sizable decreases in reported depression between baseline and post-intervention. Although intervention group participants reported less depressive symptoms overall between baseline and post-post intervention, they did report a slight increase in depressive symptomatology between post-intervention and post-post intervention.

Effect sizes between workshop participation and depressive symptoms at post-intervention and post-post intervention were large. When comparing baseline scores to those after workshop participation, there was a clear effect of participation at post-intervention (*d* =1.63) and post-post intervention (*d* =1.68).

When looking at the association between depressive symptoms and functionality (Table 3), depressive symptoms had moderate effects upon social/role limitations and nights spent in the hospital. As levels of depression increase, there was a moderate positive association to reported social/role limitations (*r* =0.40*). In addition, there was also a moderate negative relation between increased depression and nights spent in the hospital (*r* =−0.38) and exercise behaviors (*r* =−0.39).

### Workshop participation and functionality

Workshop participation had a strong negative effect upon social/ role limitations (*r* =−0.62**) and pain (*r* =−0.45*). Participation in the workshop was associated with less social role/limitations and less pain (Table 3). Workshop participation was also strongly correlated with exercise behaviors with workshop participation being positively related to increased reported exercise (*r* =0.63**).

## Discussion

Not only did the larger pilot project demonstrate sizable gains in stress and depression as a result of workshop participation; this nested study also showed that those gains were correlated/positively associated with improved health behaviors. While the current study was underpowered to detect statistically significant differences, findings could have implications for disease experience and quality of life in the population most affected by this disease.

Patients’ inability to cope may negatively affect their ability to make rational informed decisions regarding their health care as well as daily disease maintenance activities. SLE patients who are unable to cope with the stressors of their daily lives may develop an avoidant coping style that may manifest as self-blaming, depression, “wishful thinking”, and physical/social disability [38]. These dysfunctional coping styles can be reduced over the long-term when patients learn appropriate coping mechanisms [39]. As previously mentioned, physiological stress has a direct affect on the development and severity of SLE and other autoimmune diseases [1]. The severity of the disease, including the degree of pain, the extent of physical disability, and emotional despair also has an effect on the level of physiological distress the patient may experience [40]. Patients dealing with active SLE are at greater risk of having physiological difficulties such as major depression [41]. Whether physiological stress has negative effects on SLE or SLE has negative effects on the physiological well-being of the patient, it is important that SLE patients develop and master coping skills that help them better manage difficult situations and circumstances in-turn improving their quality of life.

Even with our study’s statistical uncertainty, recognizing the positive relationship between physiological stress and active SLE reinforces the importance of interventions that better equip patients with the appropriate coping skills that have the potential to improve their quality of life. Psychological interventions improve SLE patients’ mental health status [39].

Overall, our results support the importance of addressing psychosocial factors in SLE disease management. It seems that pain, fatigue, self efficacy, and depression go together and suggest that treating the disease alone does not adequately treat the underlying psychosocial elements that affect patients’ ability to cope with and manage disease [42–50]. The clinical observation is that if the patient is depressed and unable to cope, they don’t tend to improve [51,52]. Therefore, one potential method of improving outcomes in African- American lupus patients would be to address disease activity and depression simultaneously during clinic visits.

## Figures and Tables

**Table 1:** Characteristics of study participants.

Characteristic	Treatment Group (N=15)	Control Group (N=15)
Mean ± SD age, years	43.4 ± 11.7	42.1 ± 12.3
No. (%) African American	15 (100)	15 (100)
No. (%) female	14 (93)	14 (93)
No. (%) attended trade school*	2 (15)	6 (46)
No. (%) attended college*	4 (31)	4 (31)

* Four participants (two from each group) did not complete information on education level.

**Table 2:** Mean Differences Scores between Baseline and Post-Intervention by Group.

Variable	Post-Intervention* (N=25)			4 Months* (N=22)		
	*Mint*	*Mcon*	*d*	*Mint*	*Mcon*	*d*
Lupus Self-Efficacy	19.17	−3.21	0.85	9.67	−1.67	0.46
State-Trait Anxiety Inventory	0.58	−1.81	0.31	1.13	−0.33	0.25
Beck Depression Inventory II	−7.21	2.89	1.63	−4.5	2.08	1.68
Cognitive Symptom Management	0.92	0.33	0.45	0.85	0.33	0.47
Health Distress	−0.94	0.31	0.94	−0.5	0.33	0.73

* *Mint*=mean difference in intervention group; *Mcon*=mean difference in control group

**Table 3:** Correlation Matrix of Difference Scores between Baseline and Post-Intervention.

	LSES	STAI	BDI-II	CSM	HD	GROUP
Social/Role Limitations	−0.36	−0.24	0.40*	−0.38	0.50*	−0.62**
Adapted Illness Intrusiveness	0.07	0.48*	0.05	−0.15	0.18	−0.07
Communication w/Physicians	0.14	0.04	−0.02	0.25	−0.11	0.13
Visits to Hospital	−0.33	−0.24	−0.01	−0.14	0.26	−0.31
Nights Spent in Hospital	0.1	−0.27	−0.38	0.01	0.16	0.08
Pain	−.40*	−0.06	0.25	−0.2	0.66**	−0.45*

* *p*<0.05;

** *p*<0.01
